# Diabetes mediates an inverted L-shaped association between cardiometabolic index and kidney stones: a cross-sectional study from NHANES 2007-2020

**DOI:** 10.3389/fendo.2025.1535724

**Published:** 2025-02-25

**Authors:** Jinghui Bi, Jianwei Du, Xiaoyi Yan, Rongxin Chen

**Affiliations:** ^1^ Department of Urology, Liaocheng People’s Hospital, Liaocheng, Shandong, China; ^2^ Department of Critical Care Medicine, Weifang People’s Hospital, Shandong Second Medical University, Weifang, Shandong, China; ^3^ Department of Health, Weifang People’s Hospital, Shandong Second Medical University, Weifang, Shandong, China; ^4^ Department of Urology, Shengli Oilfield Central Hospital, Dongying, Shandong, China

**Keywords:** cardiometabolic index, kidney stone, diabetes, NHANES, cross-sectional

## Abstract

**Background:**

Kidney stones are a chronic metabolic disorder. The cardiometabolic index (CMI) is a new and easily accessible measure used to assess metabolic status. However, the relationship between CMI and the incidence of kidney stones remains unclear.

**Methods:**

Data from the National Health and Nutrition Examination Survey (NHANES) was used in our cross-sectional study. A weighted multivariable logistic regression analysis was conducted to assess the relationship between CMI and kidney stone incidence. Subgroup and restricted cubic spline regression analyses were utilized to confirm robustness and assess the non-linearity of the association between CMI and kidney stone incidence.

**Results:**

This study involved 18,043 individuals, of whom 9.89% were diagnosed with kidney stones. After controlling for all covariates, CMI showed a significant positive association with kidney stone incidence (OR: 1.07, 95%CI: 1.02-1.12). Individuals in the highest CMI quartile experienced a 50% higher incidence of kidney stones than those in the lowest quartile (OR: 1.50, 95%CI: 1.18-1.92). Additionally, a significant interaction was observed in the subgroup with a history of diabetes (p < 0.05).

**Conclusion:**

The study identified a notable non-linear relationship between elevated CMI levels and a greater occurrence of kidney stones. This finding suggests that by routinely monitoring CMI levels, physicians can identify individuals at risk for kidney stones early, allowing for timely intervention to mitigate disease progression.

## 1 Introduction

Kidney stones, a chronic metabolic disorder, are predominantly characterized by the dysregulation of salt dissolution and precipitation within urine (1, 2). Empirical research has delineated obesity, diabetes, and hypertension as the principal risk factors contributing to stone formation (3). Over the last 50 years, there has been a notable rise in the occurrence of kidney stones. The National Health and Nutrition Examination Survey (NHANES) indicates that the rate of self-reported kidney stones in the U.S. has almost tripled over three decades, increasing from 3.2% between 1976 and 1980 to 8.8% between 2007 and 2010 (4, 5). Similarly, in the United Kingdom, a 63% increase in kidney stone prevalence was observed over a decade, rising from 7.14% in 2000 to 11.62% in 2010 (6). The recurrence rate of kidney stones is notably high, with estimates suggesting a 5-year recurrence rate of up to 50% (7). Furthermore, the economic burden of kidney stones is substantial, with forecasts suggesting that the annual expenditure on kidney stone treatment in the US could surpass $4 billion by 2030 (8). The diagnosis of kidney stones is often elusive and can only be ascertained upon the expulsion, extraction, or radiographic detection of stones within the urinary tract (2). Given the subtle onset and high recurrence rate of kidney stones, the identification of effective biomarkers is imperative for prevention and economic mitigation.

The Cardiometabolic Index (CMI), initially proposed by Ichiro Wakabayashi in 2015 (9). is an innovative marker combining waist-to-height ratio (WHtR), high-density lipoprotein cholesterol (HDL-C), and triglycerides (TG), offering a strong indicator for cardiometabolic risk assessment (10). Further investigations have identified connections between CMI and a range of metabolic disorders, including diabetes, cardiovascular diseases like atherosclerosis and hypertension, and obesity, which are closely related to the formation of kidney stones (11–13). Nonetheless, the relationship between CMI and the occurrence of kidney stones is not well-studied and requires more research.

This research sought to examine the connection between the CMI and the frequency of kidney stones using data from NHANES. The goal is to offer an efficient and accessible biomarker for kidney stone prevention and treatment.

## 2 Methods

### 2.1 Study population

The research data is sourced from NHANES, a national cross-sectional study evaluating the nutritional and health status of Americans (14). Comprehensive information is available on the official NHANES website (https://www.cdc.gov/nchs/nhanes). All NHANES studies were approved by the NCHS Research Ethics Review Board, with participants giving written informed consent (15).

**Figure 1:**
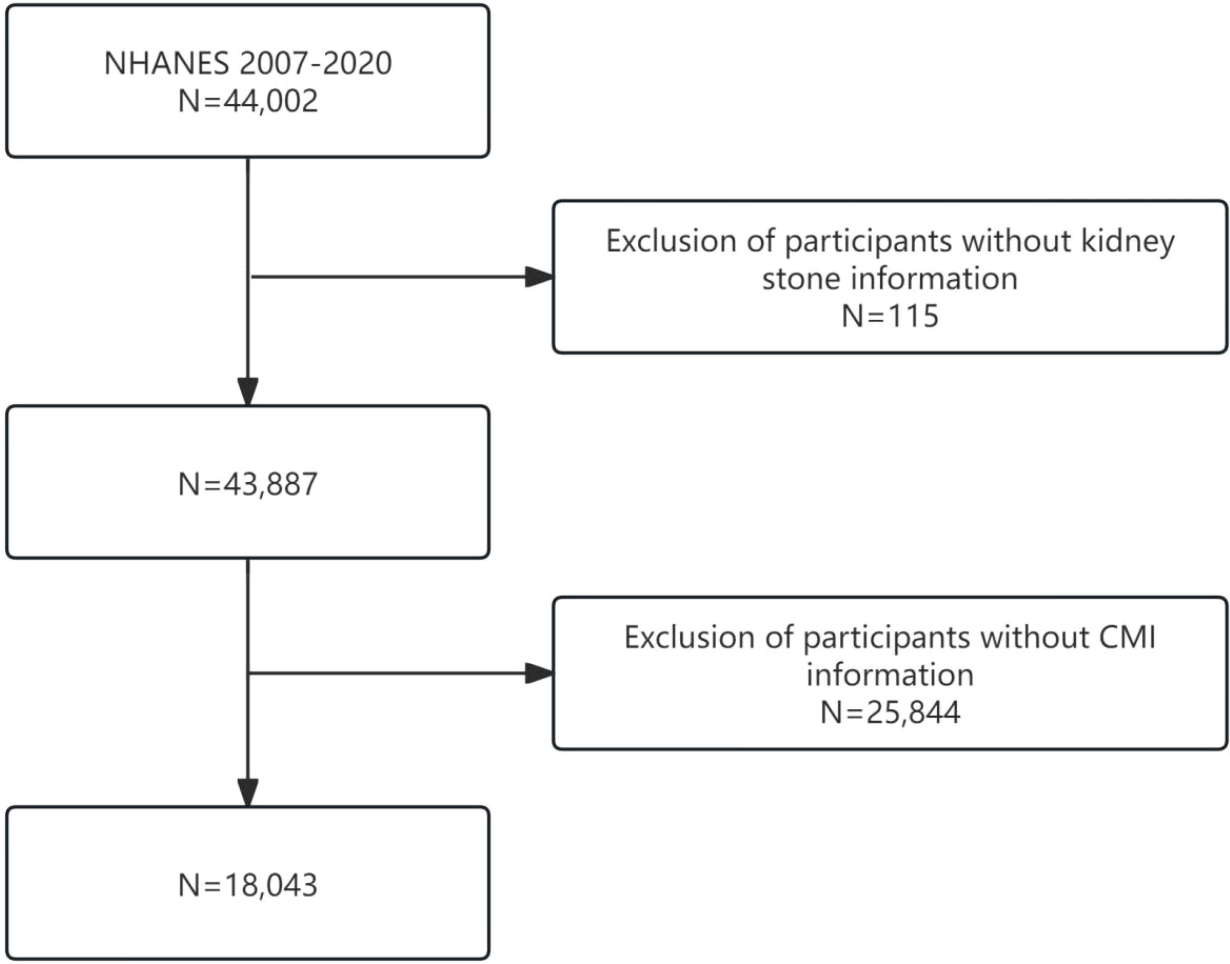
Flowchart of our subjects.

This study analyzes data from seven consecutive cycles of the NHANES conducted between 2007 and 2020. The data collection initially comprised 44,002 participants. However, after excluding individuals with missing information on kidney stones (n = 115), the CMI (n = 25,844), and other relevant covariates (n = 7,801), the final individuals consisted of 18,043 participants. A flow chart illustrating the participant inclusion criteria is presented in **Figure 1**.

### 2.2 Outcome and exposure

The study’s main outcome measure is the incidence of kidney stones, assessed through participants’ responses to the Kidney Status Questionnaire (KSQ) item, “are you ever had kidney stones?” (variable KIQ026). Participants who answered “yes” were classified as having a history of kidney stones.

The CMI is calculated using the following formula: CMI = TG (mmol/L)/HDL-C (mmol/L) × waist circumference (cm)/height (cm) (9).

### 2.3 Covariates

This study included several covariates: age, sex, race, poverty-income ratio (PIR), marital status, education level, body mass index (BMI), alcohol consumption, smoking, physical activity, diabetes, and hypertension. Sex was classified as either male or female. Race was categorized as Mexican American, other Hispanic, non-Hispanic white, non-Hispanic black, and other races. Education levels were categorized into three groups: less than high school, high school, and above high school. Marriage was categorized as married, never married, and widowed. Household income was evaluated using the PIR index, representing the ratio of household income to the poverty threshold, adjusted for household size (16). BMI was determined by dividing weight in kilograms by the square of height in meters. Smoking is categorized into three groups: former smokers, who have smoked over 100 cigarettes in their lifetime but do not currently smoke; never smokers, who have smoked fewer than 100 cigarettes; and current smokers, who are actively smoking (17). Alcohol consumption is categorized according to drinking habits: non-drinkers (never drank or quit in the past year), moderate drinkers (< 2 cups/day for men and < 1 cup/day for women), or heavy drinkers (≥ 2 cups/day for men and ≥ 1 cup/day for women) (18).

The 2024 American Diabetes Association (ADA) standards define diabetes through the following criteria: 1) self-reported diagnosis, 2) insulin or oral hypoglycemic medication use, 3) random blood glucose ≥ 11.1 mmol/L, 4) glycated hemoglobin ≥ 6.5%, 5) fasting blood glucose (FPG) ≥ 7.0 mmol/L, or 6) two-hour oral glucose tolerance test (OGTT) blood glucose ≥ 11.1 mmol/L (19). Participants were identified as having high blood pressure if they confirmed being diagnosed by a healthcare professional or reported current use of prescription medication for high blood pressure (20). The American Heart Association guidelines define high blood pressure by averaging systolic and diastolic blood pressure from three measurements taken at rest (21). Participants are classified as having high blood pressure if their systolic blood pressure (SBP) is 140 mmHg or higher and/or their diastolic blood pressure (DBP) is 90 mmHg or higher. The activity was classified into sedentary, moderate (exercising for at least 10 minutes in the past 30 days, causing light sweating or breathing), or vigorous (exercising for at least 10 minutes in the past 30 days, causing heavy sweating or an increased heart rate) (22).

### 2.4 Statistical analysis

The participants were divided into four groups according to CMI quartiles: Q1 < 0.091, 0.091 ≤ Q2 < 0.137, 0.137 ≤ Q3 < 0.210, and Q4 ≥ 0.210. Continuous variables are shown as averages with standard errors, and categorical variables are represented by frequencies and percentages. Differences in baseline characteristics were calculated using chi-square tests for categorical variables and t-tests or one-way ANOVAs for continuous variables. Three distinct multivariable logistic regression models were used to examine the relationship between CMI and kidney stone incidence. Model 1 did not include any covariate adjustments. Model 2 included adjustments for age, sex, race, and PIR. Model 3 included factors such as age, sex, PIR, race, education, marriage, hypertension, diabetes, alcohol consumption, smoking, and activity. Furthermore, to confirm the robustness and nonlinearity of the association between CMI and kidney stones, we conducted subgroup analyses and restricted cubic spline (RCS) regression analyses.

R software (version 4.3.3) was used for the analyses, with statistical significance determined by a p-value of less than 0.05.

## 3 Results

### 3.1 Participant characteristics

**Table 1** delineates the baseline characteristics of participants enrolled in the NHANES from 2007 to 2020, categorized by CMI quartiles. The study analyzed 18,034 individuals, with an average age of 47.73 ± 0.24 years. Females comprised 51.50% of the participants, and the prevalence of kidney stones was 9.89%. Participants in Q4 were generally older, predominantly male, non-Hispanic white, had lower income levels, and were more likely to be married compared to those in Q1. They also demonstrated higher educational levels and BMI. Additionally, this group exhibited moderate alcohol intake, non-smoking habits, decreased physical activity, and a higher occurrence of diabetes, hypertension, and kidney stones (p < 0.05).

**Table 1:** Baseline character of our subjects grouped by CMI quartile.

	Total (n=18043)	Q1 (n=4464)	Q2 (n=4515)	Q3 (n=4541)	Q4 (n=4523)	P
Age, years	47.73(0.24)	43.75(0.43)	47.32(0.37)	48.90(0.31)	51.16(0.34)	<0.001
Sex, n (%)						<0.001
Female	9295(51.50)	2602(59.31)	2358(52.08)	2292(50.74)	2043(43.51)	
Male	8748(48.50)	1862(40.69)	2157(47.92)	2249(49.26)	2480(56.49)	
Race, n (%)						<0.001
Mexican American	2682(8.72)	373(5.34)	619(8.36)	805(10.34)	885(11.06)	
Non-Hispanic Black	3809(10.51)	1407(15.73)	1095(11.69)	837(9.36)	470(4.99)	
Non-Hispanic White	7175(66.37)	1601(64.35)	1728(66.54)	1772(64.71)	2074(69.92)	
Other Hispanic	1954(6.13)	373(5.46)	461(5.72)	566(7.20)	554(6.20)	
Other Race	2423(8.26)	710(9.12)	612(7.68)	561(8.40)	540(7.83)	
PIR	3.01(0.04)	3.22(0.04)	3.06(0.05)	2.87(0.05)	2.87(0.05)	<0.001
Marriage, n (%)						<0.001
Never married	3929(18.13)	824(15.33)	986(18.44)	1055(19.39)	1064(19.53)	
Married	10840(64.11)	2490(61.68)	2691(63.73)	2803(64.65)	2856(66.59)	
Widowed	3265(17.73)	1148(22.99)	836(17.83)	683(15.96)	598(13.88)	
Education, n (%)						<0.001
Less than high school	1780(5.19)	240(3.14)	348(4.21)	574(6.46)	618(7.11)	
High school	6586(33.35)	1441(27.97)	1638(32.07)	1732(37.17)	1775(36.59)	
More than high school	9661(61.42)	2779(68.89)	2526(63.72)	2230(56.36)	2126(56.30)	
CMI	0.17(0.00)	0.07(0.00)	0.11(0.00)	0.17(0.00)	0.33(0.00)	<0.001
BMI, kg/m^2^	29.08(0.09)	24.75(0.10)	27.91(0.12)	30.47(0.14)	33.46(0.17)	<0.001
Alcohol consumption, n (%)						<0.001
No	4079(18.98)	840(17.07)	934(19.64)	1069(22.71)	1236(25.58)	
Moderate	8262(51.36)	2279(63.75)	2159(57.91)	2001(54.18)	1823(53.03)	
Heavy	3299(19.27)	744(19.18)	838(22.45)	854(23.12)	863(21.38)	
Smoking, n (%)						<0.001
Former	4373(25.55)	874(22.16)	998(23.79)	1139(25.81)	1362(30.68)	
Never	10081(55.45)	2815(61.96)	2586(56.87)	2478(53.47)	2202(49.23)	
Now	3575(18.96)	773(15.88)	923(19.34)	921(20.72)	958(20.10)	
Diabetes, n (%)						<0.001
No	13948(82.71)	4101(95.15)	3809(90.42)	3383(82.31)	2655(65.47)	
Yes	3921(16.31)	324(4.85)	667(9.58)	1104(17.69)	1826(34.53)	
Hypertension, n (%)						<0.001
No	10545(63.02)	3283(78.45)	2779(66.72)	2475(59.26)	2008(46.86)	
Yes	7494(36.96)	1180(21.55)	1735(33.28)	2064(40.74)	2515(53.14)	
Kidney stone, n (%)						<0.001
No	16318(90.11)	4188(93.46)	4129(90.90)	4062(89.26)	3939(86.63)	
Yes	1725(9.89)	276(6.54)	386(9.10)	479(10.74)	584(13.37)	
Activity, n (%)						<0.001
Sedentary	9445(46.04)	1906(35.37)	2234(43.96)	2556(50.31)	2749(55.12)	
Moderate	4645(28.32)	1096(26.00)	1220(29.00)	1162(28.91)	1167(29.49)	
Vigorous	3953(25.64)	1462(38.63)	1061(27.05)	823(20.78)	607(15.39)	

Data were n (%) or mean ± standard error; CMI, cardiometabolic index; PIR, poverty income ratio; BMI, body mass index.

CMI quartiles: Q1<0.091, 0.091≤Q2<0.137, 0.137≤Q2<0.210, and 0.210≤Q4.

**Table 2** presents the baseline demographic characteristics of participants categorized by their history of kidney stones. Patients with a history of kidney stones tended to be older, male, Non-Hispanic White, married, and exhibited higher BMI and CMI. They also showed moderate alcohol consumption, were never smokers, had a history of hypertension, were non-diabetic, and engaged in reduced physical activity (p < 0.05).

**Table 2:** Baseline characteristics among groups according to kidney stone status.

	Total	Non-kidney stone	Kidney stone	P
Age, years	47.73(0.24)	47.12(0.24)	53.28(0.47)	<0.001
Sex, n (%)				<0.001
Female	9295(51.50)	8515(52.08)	780(46.26)	
Male	8748(48.50)	7803(47.92)	945(53.74)	
Race, n (%)				<0.001
Mexican American	2682(8.72)	2463(8.96)	219(6.58)	
Non-Hispanic Black	3809(10.51)	3598(11.10)	211(5.14)	
Non-Hispanic White	7175(66.37)	6278(65.43)	897(74.93)	
Other Hispanic	1954(6.13)	1748(6.16)	206(5.87)	
Other Race	2423(8.26)	2231(8.35)	192(7.48)	
PIR	3.01(0.04)	3.01(0.04)	3.01(0.06)	0.991
Marriage, n (%)				<0.001
Never married	3929(18.13)	3477(17.81)	452(21.09)	
Married	10840(64.11)	9751(63.67)	1089(68.31)	
Widowed	3265(17.73)	3082(18.52)	183(10.60)	
Education, n (%)				0.894
Less than high school	1780(5.19)	1603(5.18)	177(5.35)	
High school	6586(33.35)	5956(33.32)	630(33.77)	
More than high school	9661(61.42)	8744(61.51)	917(60.88)	
BMI, kg/m^2^	29.08(0.09)	28.89(0.09)	30.84(0.22)	<0.001
CMI	0.17(0.00)	0.16(0.00)	0.20(0.00)	<0.001
Alcohol consumption, n (%)				0.001
No	4079(18.98)	3647(20.70)	432(25.66)	
Moderate	8262(51.36)	7500(57.37)	762(56.84)	
Heavy	3299(19.27)	3036(21.93)	263(17.50)	
Smoking, n (%)				0.010
Former	4373(25.55)	3867(25.09)	506(29.85)	
Never	10081(55.45)	9197(55.93)	884(51.28)	
Now	3575(18.96)	3241(18.98)	334(18.87)	
Diabetes, n (%)				<0.001
No	13948(82.71)	12817(84.69)	1131(73.08)	
Yes	3921(16.31)	3332(15.31)	589(26.92)	
Hypertension, n (%)				<0.001
No	10545(63.02)	9756(64.56)	789(49.11)	
Yes	7494(36.96)	6558(35.44)	936(50.89)	
Activity, n (%)				<0.001
Sedentary	9445(46.04)	8439(45.30)	1006(52.73)	
Moderate	4645(28.32)	4205(28.24)	440(29.08)	
Vigorous	3953(25.64)	3674(26.46)	279(18.19)	

Data were n (%) or mean ± standard error; CMI, cardiometabolic index; PIR, poverty income ratio; BMI, body mass index.

### 3.2 Correlation between CMI and kidney stones

**Table 3** shows the relationship between CMI and kidney stone incidence. Treating CMI as a continuous variable revealed a significant positive correlation with kidney stone incidence in Model 1 (OR: 1.17; 95%CI: 1.12-1.21, p < 0.001). This association remained statistically significant after adjusting for confounding factors (OR: 1.07; 95%CI: 1.02-1.12, p = 0.006). Additionally, when CMI was categorized into quartiles, the results indicated that in Model 3, as CMI levels increased, the ORs of kidney stones were 1.32, 1.44, and 1.50, respectively. This finding suggests that higher CMI levels correspond to an increased incidence of kidney stones (p for trend = 0.001), confirming the statistical significance of this upward trend. Both Model 1 and Model 2 exhibited similar patterns (p for trend < 0.001).

**Table 3:** The relationship between CMI and kidney stone risk.

	Model 1		Model 2		Model 3	
	OR (95%CI)	P	OR (95%CI)	P	OR (95%CI)	P
10-folds CMI	1.17(1.12,1.21)	<0.001	1.12(1.08,1.17)	<0.001	1.07(1.02,1.12)	0.006
CMI quartiles						
Q1	ref		ref		ref	
Q2	1.43(1.15,1.79)	0.002	1.33(1.06,1.68)	0.015	1.32(1.01,1.73)	0.042
Q3	1.72(1.41,2.10)	<0.001	1.50(1.22,1.85)	<0.001	1.44(1.13,1.82)	0.003
Q4	2.21(1.82,2.67)	<0.001	1.82(1.49,2.23)	<0.001	1.50(1.18,1.92)	0.001
P for trend		<0.001		<0.001		0.001

Model 1: no adjustment.

Model 2: adjusted for age, sex, PIR, and race.

Model 3: adjusted for age, sex, PIR, race, education, marriage, hypertension, diabetes, alcohol consumption, smoking, and activity.

### 3.3 RCS regression

**Figure 2:**
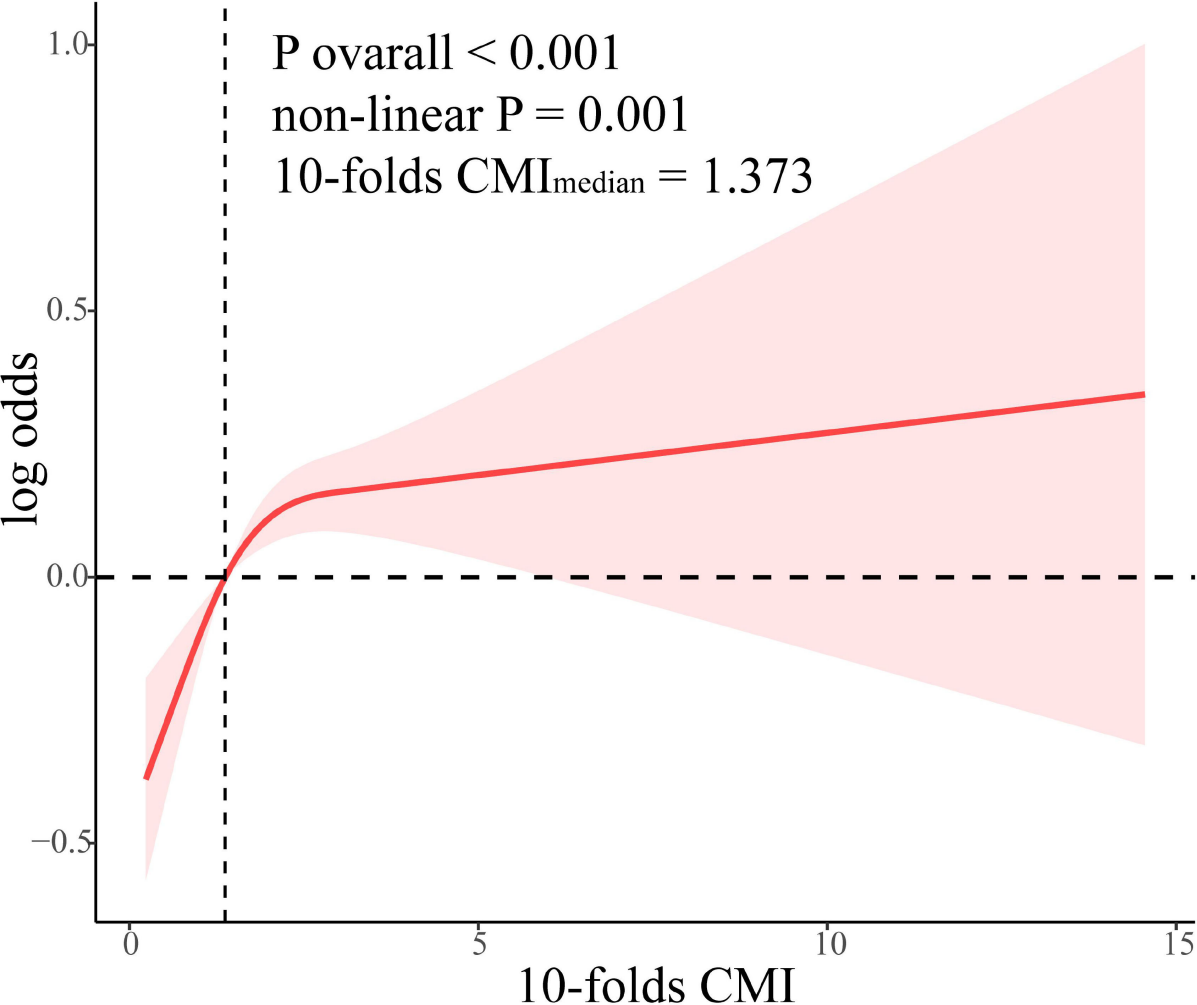
Dose-relationship between CMI and kidney stone.

A dose-response relationship between CMI and kidney stone prevalence was examined using an RCS analysis, revealing a notable nonlinear connection (p non-linear = 0.001). An increase in CMI significantly raises the prevalence of kidney stones. Specifically, the incidence of kidney stones initially rises quickly with increasing CMI levels, and then tends to remain somewhat constant, or increases at a much slower rate once the CMI exceeds 1.373 (**Figure 2**).

### 3.4 Subgroup analyses

Additional subgroup analyses were conducted to assess the impact of different factors on the association between CMI and kidney stone incidence. The factors considered were age, sex, BMI, smoking, alcohol consumption, and diabetes. The findings indicated that significant interactions were only observed for diabetes, with no other interactions reaching significance (p for interaction > 0.05). Age, gender, BMI, smoking, and alcohol consumption did not significantly influence the positive correlation between CMI and kidney stones (p > 0.05). A significant positive association was found in the diabetes subgroup (OR: 1.88, 95%CI: 1.29-2.74, *p* = 0.001), whereas the non-diabetes subgroup showed no significant association (p > 0.05) (**Table 4**).

**Table 4:** Subgroup analysis of the relationship between CMI and kidney stone risk.

	CMI < 1.373	CMI ≥ 1.373	P	P for interaction
Age				0.790
<60	ref	0.99(0.76,1.28)	0.923	
≥60	ref	1.27(0.91,1.76)	0.163	
Sex				0.999
male	ref	1.07(0.77,1.48)	0.682	
female	ref	1.07(0.80,1.43)	0.636	
BMI				0.147
normal	ref	1.45(0.92,2.27)	0.107	
overweight	ref	0.97(0.70,1.36)	0.869	
obese	ref	0.95(0.68,1.33)	0.779	
Smoking				0.516
never	ref	1.11(0.84,1.47)	0.475	
former	ref	0.93(0.67,1.29)	0.666	
now	ref	1.111(0.745,1.657)	0.601	
Alcohol consumption				0.092
no	ref	1.00(0.71,1.42)	0.996	
moderate	ref	0.98(0.77,1.26)	0.897	
heavy	ref	1.49(0.95,2.33)	0.084	
Diabetes				0.001
no	ref	0.95(0.76,1.18)	0.627	
yes	ref	1.88(1.29,2.74)	0.001	

The P-value for the interaction test was calculated from the likelihood ratio test.

All results were calculated by adjusting the covariates in Model 3 (except for the subgroups themselves).

## 4 Discussion

This research uniquely explores the link between CMI and kidney stone incidence utilizing NHANES data from 2007 to 2020. The findings demonstrate that individuals with kidney stones had elevated CMI levels relative to those without. A significant positive correlation between CMI and kidney stone incidence was observed after adjusting for confounding factors. Additionally, smooth curve fitting analysis revealed the nonlinear relationship between CMI and kidney stones, with an overall upward trend. This finding indicates that CMI could be a useful marker for evaluating kidney stone risk. Finally, subgroup analyses showed that diabetes mediated this positive association.

The CMI integrates obesity and blood lipid levels (WHtR, TG, and HDL-C), serving as a valuable new indicator for assessing diabetes risk (9). Prior studies have shown a correlation between CMI and multiple diseases. Yan et al. examined 2,996 participants, identifying a significant positive correlation between CMI and non-alcoholic fatty liver disease (NAFLD), with a predictive area under the curve of 0.762 (23). Liu et al. identified a significant correlation between body adiposity index (BAI), conicity index (CI), body shape index (ABSI), body roundness index (BRI), visceral adipose index (VAI), and lipid accumulation products (LAP) after adjusting for confounding variables, with a stronger correlation observed between CMI and hyperuricemia (24). A similar study found a positive correlation between CMI and depression, indicating that each unit increase in CMI corresponded to a 36% higher risk of depressive symptoms in the fully adjusted model (10). Notably, patients in the highest CMI quartile exhibited a 62% higher risk of depression compared to participants in the lowest quartile. Likewise, CMI is associated with other conditions, including obstructive sleep apnea and stroke (25, 26). This study examines the link between kidney stone incidence and CMI level variations. The study indicates a significant positive correlation between CMI and kidney stone incidence, even after adjusting for confounding factors, whether CMI is considered a continuous or categorical variable. This study advances the comprehension of risk factors linked to kidney stone formation.

Recent research highlights metabolic disorders as key factors in kidney stone formation (27). These disorders, which encompass overweight/obesity, insulin resistance, diabetes, and dyslipidemia, are particularly associated with abnormalities in lipid metabolism that may facilitate kidney stone development (28–30). Several investigations have demonstrated that dysregulated lipid metabolism can promote stone formation by altering urinary levels of calcium and oxalate, as well as urinary PH (27, 31). This study employed the NHANES database to investigate the link between CMI and kidney stones, identifying a significant positive correlation with the incidence of kidney stones. However, subgroup analyses indicated notable dependence on diabetes and hypertension in this positive relationship, suggesting that the association is more pronounced in patients with these conditions. Torricelli et al. identified a link between dyslipidemia and a heightened risk of nephrolithiasis, independent of other metabolic syndrome factors such as diabetes and obesity (13).

Our study revealed that patients in the kidney stones group exhibited elevated CMI at baseline. To deepen our understanding of the link between lipid parameters and kidney stones and to strengthen the connection to clinical practice, we reviewed prior studies examining the associations between different lipid indices and kidney stones. The WHtR effectively accounts for abdominal obesity and body shape, serving as a low-cost, simple, and efficient measurement index (32). It outperforms BMI and waist circumference in assessing abdominal fat for screening cardiometabolic risk factors (33). Obesity is a recognized independent risk factor for kidney stones (34). Taylor et al. discovered that greater waist circumference and/or higher weight elevated the likelihood of developing kidney stones, with the heightened risk possibly being more significant in women compared to men (35). Furthermore, previous studies have indicated that obesity rates are rising more rapidly among female patients compared to their male counterparts, suggesting that changes in obesity may account for the gender differences observed in kidney stone prevalence (36). Our study found a higher prevalence of kidney stones in women than in men, possibly due to hormonal or lifestyle factors unique to women, contrasting with previous findings. It is recognized as a protective factor against coronary heart disease due to its role in facilitating the removal of excess cholesterol from atherosclerotic plaques and transporting it back to the liver for excretion (37). Gao et al. identified a correlation between low HDL-C levels and a heightened risk of kidney stones in US adults (38). This leads to the hypothesis that decreased HDL-C levels may influence renal blood flow and the calcification process, potentially promoting kidney stone formation. Notably, urinary stones are characterized by elevated levels of TG. Yao et al. utilized the NHANES database to demonstrate that higher TG levels are independently linked to an increased incidence of kidney stones (39). Therefore, enhancing health education for patients with obesity or lipid abnormalities, along with effective management of blood lipid levels, may help reduce the formation of kidney stones and improve overall health.

However, this study has several limitations. As a cross-sectional study, it cannot determine a causal link between CMI and kidney stone incidence. Second, while we have controlled for and eliminated many confounding factors, there may still be additional confounders that could influence the analysis. Furthermore, although this study was conducted on a nationwide scale, the data were primarily derived from America, which may impact the representativeness and generalizability of the findings due to potential sample selection bias. This study identifies potential biomarkers for clinicians by elucidating the link between CMI, lipid metabolism, obesity, and kidney stone risk. This insight can inform adjustments to treatment plans and interventions based on patients’ CMI levels; however, further prospective studies are required to validate these findings.

## 5 Conclusion

The research shows a notable non-linear connection between CMI and the occurrence of kidney stones, with higher CMI levels linked to more frequent kidney stone cases. Additionally, there is a more notable relationship between CMI and kidney stones in diabetic individuals.

## Data Availability

The raw data supporting the conclusions of this article will be made available by the authors, without undue reservation.

## References

[1] Gao H Lin J Xiong F Yu Z Pan S Huang Y. Urinary microbial and metabolomic profiles in kidney stone disease. Front Cell infection Microbiol. (2022) 12:953392. doi: 10.3389/fcimb.2022.953392 PMC9484321 36132987

[2] Khan SR Pearle MS Robertson WG Gambaro G Canales BK Doizi S. Kidney stones. Nat Rev Dis Primers. (2016) 2:16008. doi: 10.1038/nrdp.2016.8 27188687 PMC5685519

[3] Hao X Shao Z Zhang N Jiang M Cao X Li S. Integrative genome-wide analyses identify novel loci associated with kidney stones and provide insights into its genetic architecture. Nat Commun. (2023) 14:7498. doi: 10.1038/s41467-023-43400-1 37980427 PMC10657403

[4] Stamatelou KK Francis ME Jones CA Nyberg LM Curhan GC. Time trends in reported prevalence of kidney stones in the United States: 1976-1994. Kidney Int. (2003) 63:1817–23. doi: 10.1046/j.1523-1755.2003.00917.x 12675858

[5] Scales CD Jr. Smith AC Hanley JM Saigal CS. Urologic Diseases in America, Prevalence of kidney stones in the United States. Eur Urol. (2012) 62:160–5. doi: 10.1016/j.eururo.2012.03.052 PMC3362665 22498635

[6] Turney BW Reynard JM Noble JG Keoghane SR. Trends in urological stone disease. BJU Int. (2012) 109:1082–7. doi: 10.1111/j.1464-410X.2011.10495.x 21883851

[7] Fink HA Wilt TJ Eidman KE Garimella PS MacDonald R Rutks IR. Medical management to prevent recurrent nephrolithiasis in adults: a systematic review for an American College of Physicians Clinical Guideline. Ann Intern Med. (2013) 158:535–43. doi: 10.7326/0003-4819-158-7-201304020-00005 23546565

[8] Balawender K Łuszczki E Mazur A Wyszyńska J. The multidisciplinary approach in the management of patients with kidney stone disease-A state-of-the-art review. Nutrients. (2024) 16:1932. doi: 10.3390/nu16121932 38931286 PMC11206918

[9] Wakabayashi I Daimon T. The “cardiometabolic index” as a new marker determined by adiposity and blood lipids for discrimination of diabetes mellitus. Clinica chimica acta; Int J Clin Chem. (2015) 438:274–8. doi: 10.1016/j.cca.2014.08.042 25199852

[10] Zhou X Tao XL Zhang L Yang QK Li ZJ Dai L. Association between cardiometabolic index and depression: National Health and Nutrition Examination Survey (NHANES) 2011-2014. J Affect Disord. (2024) 351:939–47. doi: 10.1016/j.jad.2024.02.024 38341157

[11] Acosta-García E Concepción-Páez M. Cardiometabolic index as a predictor of cardiovascular risk factors in adolescents. Rev salud publica (Bogota Colombia). (2018) 20:340–5. doi: 10.15446/rsap.V20n3.61259 30844007

[12] Wu L Xu J. Relationship between cardiometabolic index and insulin resistance in patients with type 2 diabetes. Diabetes Metab syndrome obesity: Targets Ther. (2024) 17:305–15. doi: 10.2147/DMSO.S449374 PMC10821666 38283637

[13] Torricelli FC De SK Gebreselassie S Li I Sarkissian C Monga M. Dyslipidemia and kidney stone risk. J Urol. (2014) 191:667–72. doi: 10.1016/j.juro.2013.09.022 24055417

[14] Fulgoni K Fulgoni VL 3rd. trends in total, added, and natural phosphorus intake in adult americans, NHANES 1988-1994 to NHANES 2015-2016. Nutrients. (2021) 13:2249. doi: 10.3390/nu13072249 34210102 PMC8308364

[15] Neuhouser ML. The importance of healthy dietary patterns in chronic disease prevention. Nutr Res (New York N.Y.). (2019) 70:3–6. doi: 10.1016/j.nutres.2018.06.002 PMC6328339 30077352

[16] Yi H Li M Dong Y Gan Z He L Li X. Nonlinear associations between the ratio of family income to poverty and all-cause mortality among adults in NHANES study. Sci Rep. (2024) 14:12018. doi: 10.1038/s41598-024-63058-z 38797742 PMC11128441

[17] Asfar T Perez A Shipman P Carrico AW Lee DJ Alcaide ML. National estimates of prevalence, time-trend, and correlates of smoking in US people living with HIV (NHANES 1999-2016). Nicotine tobacco research: Off J Soc Res Nicotine Tobacco. (2021) 23:1308–17. doi: 10.1093/ntr/ntaa277 PMC8517967 33856483

[18] Qiu Z Chen X Geng T Wan Z Lu Q Li L. Associations of serum carotenoids with risk of cardiovascular mortality among individuals with type 2 diabetes: results from NHANES. Diabetes Care. (2022) 45:1453–61. doi: 10.2337/dc21-2371 35503926

[19] ElSayed NA Baig A Bradley S Gonzalez J Haynes A McCoy RG. Introduction: standards of care in diabetes—2024 abridged for primary care professionals. Clin Diabetes. (2024) 42:181–1. doi: 10.2337/cd24-aint PMC11040011 38666198

[20] Li Y Yuan X Zheng Q Mo F Zhu S Shen T. The association of periodontal disease and oral health with hypertension, NHANES 2009-2018. BMC Public Health. (2023) 23:1122. doi: 10.1186/s12889-023-16012-z 37308938 PMC10262359

[21] Yuan M He J Hu X Yao L Chen P Wang Z. Hypertension and NAFLD risk: Insights from the NHANES 2017-2018 and Mendelian randomization analyses. Chin Med J. (2024) 137:457–64. doi: 10.1097/CM9.0000000000002753 PMC10876227 37455323

[22] Qing L Zhu Y Yu C Zhang Y Ni J. Exploring the association between dietary Inflammatory Index and chronic pain in US adults using NHANES 1999-2004. Sci Rep. (2024) 14:8726. doi: 10.1038/s41598-024-58030-w 38622145 PMC11018766

[23] Yan L Hu X Wu S Cui C Zhao S. Association between the cardiometabolic index and NAFLD and fibrosis. Sci Rep. (2024) 14:13194. doi: 10.1038/s41598-024-64034-3 38851771 PMC11162484

[24] Liu XZ Li HH Huang S Zhao DB. Association between hyperuricemia and nontraditional adiposity indices. Clin Rheumatol. (2019) 38:1055–62. doi: 10.1007/s10067-018-4374-x 30498873

[25] Cai X Hu J Wen W Wang J Wang M Liu S. Associations of the cardiometabolic index with the risk of cardiovascular disease in patients with hypertension and obstructive sleep apnea: results of a longitudinal cohort study. Oxid Med Cell Longevity. (2022) 2022:4914791. doi: 10.1155/2022/4914791 PMC9246614 35783191

[26] Li FE Luo Y Zhang FL Zhang P Liu D Ta S. Association between cardiometabolic index and stroke: A population- based cross-sectional study. Curr neurovascular Res. (2021) 18:324–32. doi: 10.2174/1567202618666211013123557 34645376

[27] Du YZ Dong QX Hu HJ Guo B Li YH Zhang J. A cross-sectional analysis of the relationship between the non-high density to high density lipoprotein cholesterol ratio (NHHR) and kidney stone risk in American adults. Lipids Health Dis. (2024) 23:158. doi: 10.1186/s12944-024-02150-9 38802797 PMC11129406

[28] Hung JA Li CH Geng JH Wu DW Chen SC. Dyslipidemia increases the risk of incident kidney stone disease in a large Taiwanese population follow-up study. Nutrients. (2022) 14:1339. doi: 10.3390/nu14071339 35405952 PMC9000795

[29] Kang HW Lee SK Kim WT Kim YJ Yun SJ Lee SC. Hypertriglyceridemia and low high-density lipoprotein cholesterolemia are associated with increased hazard for urolithiasis. J endourology. (2014) 28:1001–5. doi: 10.1089/end.2014.0135 24684546

[30] Tanaka A Node K. Associations of metabolic disorders with hypertension and cardiovascular disease: recent findings and therapeutic perspectives. Hypertension research: Off J Japanese Soc Hypertension. (2024) 47:3338–44. doi: 10.1038/s41440-024-01737-0 38811824

[31] Howles SA Thakker RV. Genetics of kidney stone disease. Nat Rev Urol. (2020) 17:407–21. doi: 10.1038/s41585-020-0332-x 32533118

[32] Martin-Calvo N Moreno-Galarraga L Martinez-Gonzalez MA. Association between body mass index, waist-to-height ratio and adiposity in children: A systematic review and meta-analysis. Nutrients. (2016) 8:512. doi: 10.3390/nu8080512 27556485 PMC4997425

[33] Muñoz-Hernando J Escribano J Ferré N Closa-Monasterolo R Grote V Koletzko B. Usefulness of the waist-to-height ratio for predicting cardiometabolic risk in children and its suggested boundary values. Clin Nutr (Edinburgh Scotland). (2022) 41:508–16. doi: 10.1016/j.clnu.2021.12.008 35016145

[34] Kim S Chang Y Yun KE Jung HS Kim I Hyun YY. Metabolically healthy and unhealthy obesity phenotypes and risk of renal stone: a cohort study. Int J Obes (2005). (2019) 43:852–61. doi: 10.1038/s41366-018-0140-z 30006578

[35] Taylor EN Stampfer MJ Curhan GC. Obesity, weight gain, and the risk of kidney stones. Jama. (2005) 293:455–62. doi: 10.1001/jama.293.4.455 15671430

[36] Wang Y Beydoun MA Min J Xue H Kaminsky LA Cheskin LJ. Has the prevalence of overweight, obesity and central obesity levelled off in the United States? Trends, patterns, disparities, and future projections for the obesity epidemic. Int J Epidemiol. (2020) 49:810–23. doi: 10.1093/ije/dyz273 PMC7394965 32016289

[37] Abdullah SM Defina LF Leonard D Barlow CE Radford NB Willis BL. Long-term association of low-density lipoprotein cholesterol with cardiovascular mortality in individuals at low 10-year risk of atherosclerotic cardiovascular disease. Circulation. (2018) 138:2315–25. doi: 10.1161/CIRCULATIONAHA.118.034273 30571575

[38] Gao M Liu M Zhu Z Chen H. The association of dyslipidemia with kidney stone: result from the NHANES 2007-2020. Int Urol Nephrol. (2024) 56:35–44. doi: 10.1007/s11255-023-03784-x 37725273

[39] Yao L Yang P. Relationship between remnant cholesterol and risk of kidney stones in U.S. Adults: a 2007-2016 NHANES analysis. Ann Med. (2024) 56:2319749. doi: 10.1080/07853890.2024.2319749 38733306 PMC11089921

